# Metabolic syndrome among adolescents in Dubai, United Arab Emirates, is attributable to the high prevalence of low HDL levels: a cross-sectional study

**DOI:** 10.1186/s12889-018-6215-x

**Published:** 2018-11-21

**Authors:** Dalia Haroun, Rola Mechli, Razan Sahuri, Safa AlKhatib, Omar Obeid, Carla El Mallah, Lesley Wood, Khulood AlSuwaidi

**Affiliations:** 1 Department of Public Health and Nutrition, College of Natural and Health Sciences, Zayed University, Dubai, United Arab Emirates; 2 Department of Nutrition and Food Science, Faculty of Agricultural and Food Sciences, American University of Beirut, Beirut, Lebanon; 3 School Health Center, Ministry of Health and Prevention, Dubai, United Arab Emirates

**Keywords:** Metabolic syndrome, Adolescents, UAE, HDL

## Abstract

**Background:**

Metabolic syndrome (MetS) describes a combination of risk factors that increase the risk of developing chronic diseases. The prevalences of MetS and its risk factors are increasing, especially in the Arab region. A cross-sectional study was carried out to assess the prevalences of MetS and its associated risk factors among adolescents in the United Arab Emirates (UAE).

**Methods:**

A total of 596 students (308 boys and 288 girls) aged 10 to 15.9 years old were recruited from 14 public secondary schools in Dubai, UAE. Anthropometric and biochemical data were measured.

**Results:**

According to the International Diabetes Federation (IDF) criteria, the prevalence of MetS was 3.7%, and it was more common among boys than girls (12 boys versus 10 girls). MetS was also more likely to be found in students who were obese or overweight than those with normal weight. The most prevalent and significant MetS risk factor was low high-density lipoprotein (HDL) cholesterol levels.

**Conclusions:**

This study indicates the importance of carrying out further investigations about the constituents of HDL and their atherogenic effects. Additionally, these results strongly recommend setting a consensus for HDL measurement, since small variations in methodologies may lead to substantial deviations in results.

## Background

Eating habits and dietary patterns are known to be important risk factors of obesity and chronic diseases. Although genetics play an important role in determining the level of body fat, food intake and fitness level are still considered the main variables that influence body weight. The worldwide obesity epidemic has paralleled an increase in the prevalence of non-communicable diseases (NCDs), and this is thought to be highly attributable to a shift towards westernized diets, which are characterized by excessive intake of refined cereals, sugars, and fats [1].

In the United Arab Emirates (UAE), there has been a strong trend towards the adoption of a “high energy-sedentary lifestyle”, which has consequently resulted in an increase in the prevalence of obesity and all obesity-related diseases over the past few decades. According to the UAE Global School Health Survey, the prevalence of overweight and obesity among Emirati adolescents dramatically increased from 21.5 to 39.2% between 2005 and 2010 [2, 3]. This alarming increase highlights the need for a public health intervention aimed at decreasing the prevalence of obesity, as excess body fat in youth is a major risk factor for obesity and metabolic abnormalities later in life [4, 5]. The data available on obesity and NCDs in UAE adults are worrisome. In 2015, the prevalence of type 2 diabetes in the UAE was 19.3% according to the International Diabetes Federation (IDF) [6]. Additionally, in 2014, NCDs were estimated to account for 65% of all deaths, and of these, 30% were attributed to only cardiovascular diseases (CVDs) according to the World Health Organization (WHO) [7]. This scenario is typical of Arab and most developing countries, especially those with higher socio-economic status [8].

It is well known that at a young age, people develop their food preferences and dietary habits and that they continue to adapt them later in life. Evidence has also shown that risk factors of obesity and NCDs can be tracked from childhood and adolescence into adulthood [9–11]. Within this context, assessments of metabolic abnormalities among young populations play an important role in predicting health risks at an older age. Metabolic syndrome (MetS) is a combination of medical disorders that includes five components: 1) elevated waist circumference; 2) increased blood pressure; 3) increased fasting glucose; 4) increased triglycerides; and 5) decreased HDL cholesterol, and the combination of these components increases the risk of developing cardiovascular diseases and diabetes [12].

A high prevalence of MetS was reported in both adults and children in the region, with higher rates found among overweight and obese subjects than in subjects with normal weight [13–17]. In UAE, the prevalence of MetS among adults is high, and extrapolation from younger ages is plausible [18]. Since Emirati citizens have various health risks [19], obesity, type 2 diabetes, and other NCDs highly prevalent among them [20–22], we aimed to determine the prevalence of MetS and its associated risk factors amongst a representative sample of children and adolescents in Dubai, UAE. Our findings would form the backbone of any programme aimed at the primary prevention and control of risk factors of chronic diseases in Dubai.

## Methods

### Study population

This is a cross-sectional study of students in grades 6 to 9 who were attending public schools in Dubai, UAE. All public schools in the UAE are sex-segregated, and the majority of the students attending these schools are Emiratis. All 14 public/governmental secondary schools in Dubai (7 schools for boys and 7 schools for girls) were included in this study. Private schools were excluded from the study as the main target population was Emirati students, who mainly attend public schools. The sample size was calculated to include approximately 10% of the total population of students attending these schools to ensure that our results are representative of Emirati adolescents. Recruitment was coordinated with the Ministry of Education, and students within schools were selected via simple random selection from a comprehensive school listing, with the student pool proportional to the school enrolment size.

Written informed consent and verbal consent were obtained from parents and participants, respectively. Students who reported having diabetes or taking any medications that affect glucose or cholesterol levels were excluded from the study. The study protocol was approved by the Zayed University Research Ethics Committee (ZU13–034-F), and recruitment was performed in collaboration with the Ministry of Education and the Ministry of Health and Prevention between March and June 2014.

With a response rate of 65 %, 596 students (308 boys and 288 girls) aged between 10 and 15.9 years old and who met the inclusion criteria were included in this cross-sectional study.

### Measurements

Personal information (date of birth, ethnicity, health problems, medication intake, etc.) and anthropometric measurements (weight, height, and waist and hip circumferences) were collected by trained field workers. Weight was measured with minimal clothing to the nearest 0.1 kg by an electronic scale (TANITA, BC-601), and height was measured without shoes to the nearest 0.1 cm using a portable stadiometer (Charder, HM-200P). Waist and hip circumferences were taken to the nearest 0.1 cm over light clothing using a non-elastic flexible tape. Waist circumference was measured at a level midway between the lowest rib and the iliac crest, and hip circumference was measured at the widest part of the buttocks. Body mass index (BMI) was calculated (weight/height^2^ (kg/m^2^)), and BMI z-scores were then plotted using the cut-offs based on the Centers for Disease Control (CDC) growth charts to classify participants into one of the following four weight categories: underweight, normal weight, overweight, and obese [23]. As no specific cut-offs were available for Emiratis, a waist circumference at or above 94 cm for boys and 80 cm for girls was categorized as a risk factor for MetS.

Students were asked to rest while seated for 5 min, during which their blood pressure and pulse rate were measured three consecutive times using a Riester machine (ri-champion N); the average of the three measurements was used. Participants were asked to fast overnight, and fasting blood samples were collected by a registered nurse. A blood analysis of glucose and a lipid profile (total cholesterol, HDL, low-density lipoprotein (LDL) and triglycerides) were then performed using a COBAS 6000 analyser (Roche Diagnostics, Switzerland).

### Definition of metabolic syndrome

The IDF criteria [6] specific for children and adolescents were used to identify MetS among subjects aged 10 to 15.9 years as follows:

- Waist circumference ≥ 94 cm for boys and ≥ 80 cm for girls (indicating abdominal obesity),
- Serum triglycerides ≥1.7 mmol/L,
- Serum HDL-cholesterol < 1.03 mmol/L,
- Serum glucose ≥5.6 mmol/L, and
- Systolic blood pressure (SBP) ≥ 130 mmHg or diastolic blood pressure (DBP) ≥ 85 mmHg.

The number of risk factors was calculated for each student, and those with a large waist circumference and at least two other risk factors were diagnosed with MetS.

### Statistical analysis

Statistical analyses were performed using SPSS version 23, and the level of significance was set at a *p*-value of < 0.05. Descriptive statistics were performed for all anthropometric and biochemical data, and values were reported as the mean ± standard deviation (SD). Categorical variables are reported as numbers and percentages. Independent sample *t*-tests were used to identify differences in all anthropometric measurements, in blood pressure and biochemical parameters between boys and girls and among the different waist circumference categories. Pearson correlations were used to examine associations between different parameters (anthropometric assessments, biochemical values and blood pressure). Logistic regression was performed to confirm the probability of having MetS. Non-parametric Chi-square tests were performed to detect differences among biochemical and waist circumference categories. Based on the Anderson–Darling normality test, two outliers were excluded for HDL levels: one for triglyceride, and two for waist circumference.

## Results

A total of 596 healthy secondary students (308 boys and 288 girls) aged between 10 and 15.9 years old were randomly selected from the public schools in Dubai. The mean age of our sample population was 13.1 ± 1.3 years old. The majority of the included students were Emiratis (83%), and the remainder were from other Arab Counties. Based on the CDC BMI categories for age/sex-related classification, 49 students (8.3%) were underweight, 308 (51.9%) were normal weight, 108 (18.6%) were overweight and 126 (21.2%) were obese. Statistical differences (*p*-value < 0.05) between boys and girls were detected for all anthropometric and body composition measurements except for body weight (*p*-value = 0.561). The boys were 3 cm taller than the girls and had lower BMI z-scores and higher waist circumferences. Fasting blood glucose and systolic blood pressure were different between boys and girls, while levels of triglycerides, total cholesterol, HDL, LDL, and diastolic blood pressure were similar (Table 1).

**Table 1:** Some of the characteristics of the sample population, separated by sex

	All (*n* = 596)	Boys (*n* = 308)	Girls (*n* = 288)	*p*-value
Anthropometric Measurements				
Age (years)	13.1 ± 1.3	13.1 ± 1.3	13.0 ± 1.3	0.498
Weight (kg)	54.6 ± 17.8	55.0 ± 20.0	54.2 ± 15.0	0.561
Height (m)	1.57 ± 0.09	1.58 ± 0.10	1.55 ± 0.64	0.038
BMI z-score	0.50 ± 1.33	0.35 ± 1.47	0.66 ± 1.15	0.005
Waist circumference (cm)	72.5 ± 11.6	73.5 ± 12.0	71.5 ± 10.9	0.029
Waist-hip ratio	0.81 ± 0.06	0.83 ± 0.05	0.78 ± 0.06	< 0.001
Blood Pressure and Biochemical Measurements				
Systolic blood pressure (mmHg)	114.9 ± 16.6	118.1 ± 19.3	111.5 ± 12.3	< 0.001
Diastolic blood pressure (mmHg)	72.0 ± 14.0	72.6 ± 16.3	71.4 ± 11.0	0.281
Glucose (mmol/L)	5.2 ± 0.4	5.24 ± 0.39	5.16 ± 0.43	0.021
Triglycerides (mmol/L)	0.79 ± 0.38	0.79 ± 0.42	0.78 ± 0.32	0.926
Cholesterol (mmol/L)	4.1 ± 0.7	4.0 ± 0.7	4.1 ± 0.7	0.122
HDL (mmol/L)	1.23 ± 0.29	1.21 ± 0.30	1.25 ± 0.28	0.111
LDL (mmol/L)	2.66 ± 0.64	2.64 ± 0.65	2.69 ± 0.62	0.357

Values are reported as the mean ± SD

T-tests were performed to detect differences in means between boys and girls. Significance was set at a *p*-value < 0.05

There was a significantly higher prevalence of borderline and high biochemical risk factors in the high waist circumference group than in the normal waist circumference group (Table 2).

**Table 2:** Prevalence of MetS-related biochemical risk factors according to waist circumference

	Total (*n* = 596)	Normal WC (*n* = 504)	High WC (*n* = 90)	*p*-value
Components of MetS^a^				
Triglycerides				
Normal (< 1.02 mmol/L)	481 (80.8)	424 (84.1)	56 (62.2)	**< 0.001**
Borderline (1.02–1.46 mmol/L)	81 (13.6)	59 (11.7)	21 (23.3)	
High (> 1.46 mmol/L)	33 (5.5)	20 (4.0)	13 (14.5)	
HDL				
Normal (> 1.17 mmol/L)	321 (54.0)	293 (58.1)	27 (30)	**< 0.001**
Borderline (1.04–1.17 mmol/L)	112 (18.9)	88 (17.5)	24 (26.7)	
Low (< 1.04 mmol/L)	161 (27.1)	121 (24.0)	39 (43.3)	
Glucose				
Normal (< 5.6 mmol/L)	503 (84.4)	426 (84.5)	75 (83.3)	**< 0.001**
Borderline (5.6–6.9 mmol/L)	93 (15.6)	78 (14.5)	15 (16.7)	
High (> 6.9 mmol/L)	0	0	0	
Non-Components of MetS^b^				
Cholesterol				
Normal (< 4.4 mmol/L)	423 (71.0)	362 (71.8)	60 (66.7)	**< 0.001**
Borderline (4.4–5.17 mmol/L)	137 (23.0)	111 (22.0)	25 (27.8)	
High (> 5.17 mmol/L)	36 (6.0)	31 (6.2)	5 (5.5)	
LDL				
Normal (< 2.85 mmol/L)	380 (63.8)	338 (67.1)	42 (46.7)	**< 0.001**
Borderline (2.85–3.34 mmol/L)	143 (24.0)	111 (22.0)	31 (34.4)	
High (> 3.34 mmol/L)	73 (12.2)	55 (10.9)	17 (18.9)	

^a^As classified by the IDF

^b^As defined by NCEP (1992) and NHANES (1994)

Values are reported as frequencies (percentages): n (%)

Non-parametric Chi-square tests were performed to detect differences among biochemical and waist circumference categories. Significance was set at a *p*-value < 0.05

N.B: some categories do not add up to 100%. This is due to missing data

Compared to students with a normal waist circumference, students with a high waist circumference had significantly higher SBP (approximately 122 mmHg versus 114 mmHg for normal waist circumference), DBP (approximately 77 mmHg versus 71 mmHg), triglycerides (0.99 mmol/L versus 0.75 mmol/L), and LDL (approximately 2.8 mmol/L versus 2.6 mmol/L). HDL means were inversely associated with waist circumference, and HDL levels were significantly higher among students with a normal waist circumference (1.25 mmol/L versus 1.1 mmol/L). All blood parameters varied according to high and normal waist circumference except glucose (approximately 5.20 mmol/L versus 5.26 mmol/L, respectively) and cholesterol levels (approximately 4.0 mmol/L versus 4.1 mmol/L, respectively).

Components of MetS were widespread among the population, with 25.4% of the students having low HDL levels (the most abundant factor among both genders) and 3.2% having high triglyceride levels. The proportions with other components ranged between two percentage points. The frequencies observed for each component of MetS are reported stratified by sex and waist circumference in Table 3.

**Table 3:** Prevalence of individual metabolic abnormalities^a^ by sex

	Boys (*n* = 308)		Girls (*n* = 288)		*p*-value
	Normal WC	High WC	Normal WC	High WC	
	*n* = 279	*n* = 27	*n* = 225	*n* = 63	
Low HDL (< 1.03 mmol/L)	14 (5.0)	13 (48.1)	39 (17.3)	24 (38.1)	0.825
High Blood Pressure					
SBP (≥130 mmHg)	16 (5.7)	11 (40.7)	56 (24.9)	7 (11.1)	0.013
DBP (≥ 85 mmHg)	20 (7.2)	7 (25.9)	59 (26.2)	4 (6.3)	0.571
High Fasting Glucose (≥5.6 mmol/L)	22 (7.9)	5 (18.5)	53 (23.6)	10 (15.9)	0.599
High Triglycerides (≥ 1.7 mmol/L)	23 (8.2)	4 (14.8)	61 (21.2)	2 (3.2)	0.066

^a^As defined by the IDF criteria for the identification of MetS

*WC* waist circumference, *SBP* Systolic Blood Pressure, *DBP* Diastolic Blood Pressure

Values are reported as frequencies (percentages): n (%)

*N.B* some categories do not add up to 100%. This is due to missing data

T-tests were performed to detect differences between boys and girls in each of the listed MetS components

The ranking (from the most to the least dominant component) differed slightly when classifications were made among students who had MetS. The most common component of MetS was low HDL levels (21 out of 22 diagnosed students, 95.5%), followed by high blood pressure (15 out of 22, 68.2%), high fasting blood glucose (9 out of 22, 40.9%), and high triglyceride levels (5 out of 22, 22.7%). Based on a logistic regression, the odds of having MetS were 65 times higher when HDL levels were low (*p*-value < 0.001), 10 times higher when blood pressure was high (*p*-value < 0.001), 7 times higher when glucose levels were elevated (*p*-value = 0.001), and 4 times higher when triglyceride levels were high (*p*-value = 0.095).

MetS was identified in 22 subjects (3.7% of the sample population) and was more prevalent in males (12 boys versus 10 girls). There was no significant difference in age (*p*-value = 0.986) between the 22 MetS students and the other students. However, students diagnosed with MetS were notably taller by approximately 7 cm (*p*-value < 0.001) and heavier by 32 kg (*p*-value < 0.001) than healthy subjects. Additionally, MetS prevalence was higher among obese 16% as compared to overweight (2%).

Subjects with a high waist circumference had significantly more risk factors. Sixty percent of individuals with a high waist circumference but only 11.6% of those with a normal waist circumference had at least two risk factors. The presence of at least four MetS risk factors was only observed in people with a high waist circumference (Table 4).

**Table 4:** Prevalence of the number of MetS risk factors by waist circumference categories

No. of MetS risk factors	Normal WC (*n* = 501)	High WC (*n* = 90)	All (*n* = 591)
0	288 (57.5)	0 (0)	288 (48.7)
1	155 (30.9)	36 (40)	191 (32.3)
2	51 (10.2)	32 (35.6)	83 (14.0)
3	7 (1.4)	18 (20)	25 (4.2)
4	0 (0)	2 (2.2)	2 (0.4)
5	0 (0)	2 (2.2)	2 (0.4)
Total	501 (84.3)	90 (15.2)	591 (100)

Values are reported as frequencies (percentages): n (%)

*WC* waist circumference

*N.B* some categories do not add up to 100% due to missing data

BMI z-scores, waist circumference, and the waist-to-hip ratio were positively and significantly correlated with blood pressure and all biochemical parameters (glucose, cholesterol, LDL and triglycerides) except cholesterol. Only HDL cholesterol was significantly inversely correlated with the variables (Table 5).

**Table 5:** Pearson correlation of anthropometric and biochemical variables

Variable	BMI z-score	WC	W:H	SBP	DBP	Glu	TG	HDL	LDL	Cl
BMI z-score	1									
WC (cm)	0.823**	1								
W:H	0.201**	0.439**	1							
SBP (mmHg)	0.349**	0.364**	0.180**	1						
DBP (mmHg)	0.198**	0.222**	0.091*	0.603**	1					
Glu (mmol/L)	0.144**	0.150**	0.154**	0.130**	0.087*	1				
TG (mmol/L)	0.237**	0.304**	0.185**	0.178**	0.104*	0.045	1			
HDL (mmol/L)	−0.301**	−0.331**	−0.169**	−0.137**	−0.067	−0.109**	− 0.334**	1		
LDL (mmol/L)	0.188**	0.194**	0.116**	0.089*	0.055	0.022	0.412**	−0.144**	1	
Cl (mmol/L)	0.080	0.079	0.062	0.050	0.043	−0.008	0.354**	0.246**	0.909**	1

*WC* waist circumference, *W:H* waist to hip ratio, *SBP* systolic blood pressure, *DBP* diastolic blood pressure, *Glu* glucose, *TG* triglycerides, *Cl* cholesterol

*p-value < 0.01; **p-value < 0.05

## Discussion

MetS is a medical condition associated with metabolic disorders. Different cut-offs have been used to define MetS, with 1) the World Health Organization [24], 2) the European Group for the Study of Insulin Resistance [25], 3) the National Cholesterol Education Program-Third Adult Treatment Panel [26], 4) the American Association of Clinical Endocrinology [27] and 5) the International Diabetes Federation [28] cut-offs being the most commonly used. The existence of several criteria has created confusion when the prevalence of MetS is compared within or among populations [28]. The IDF standard has been described as the most appropriate and the most convenient for screening MetS in children and adolescents [29] since it combines international diagnostic criteria and takes into consideration the ethnic-specific limits of waist circumference and central obesity [6]. In the UAE, the IDF standard had a sensitivity superior to those of the NCEP and ATPIII for the early diagnosis of MetS among Emirati [18, 30].

The prevalence of MetS in the region has been extensively reported among adults but has been less abundantly studied among young populations. In the UAE, the percentages have been found to vary between 29 and 40.5% [18, 19, 30] in adults, with lower but significantly high proportions also reported in children and adolescents living in the UAE [31–33]. The observed variations in numbers and percentages have been attributed to several factors, including the region selected, the reference criteria used for screening, and other important factors.

In our study, the prevalence of MetS, according to the IDF criteria, among a representative random sample of adolescents aged 10 to 15.9 years old who were attending public schools in Dubai was 3.7%. This estimate is considerably lower than those presented in previously reported results in the UAE and countries in the region, where numbers have varied significantly, mainly due to a lack of consensus on the criteria used to define MetS. Studies that have assessed the prevalence of MetS using different diagnostic standards have consistently produced different outcomes [16, 34, 35], with values sometimes differing more than two-fold [36].

Interestingly, Khashayar et al. [37] found a prevalence of 2.4% among a representative sample (5738 students) living in 23 provinces in Iran. It is worth noting that the same screening methods were used for the assessments performed in Iran, and this might have contributed to the similarities in our findings. In Egypt [17], when the NCEP was used as a reference, 7.4% of a representative sample of 4250 adolescents aged 10 to 18 years old were diagnosed with MetS, and similar results (9.1 and 9.4%) were found among adolescents living in Saudi Arabia and Kuwait (respectively) when modified ATP III criteria were used [34, 38].

Although the overall prevalence of MetS is low, its risk factors, such as abdominal obesity, were widespread among our young population, and they dominated among males. This was also demonstrated in the prevalence of MetS. Similar to other studies performed in the region [31, 36, 39–42], the prevalence of MetS was higher among boys than girls (3.9% of boys versus 3.5% of girls). This difference could be attributable to differences in sex hormones, such as testosterone and sex hormone-binding globulin (SHBG), which are strongly expressed during puberty [43].

According to BMI z-score data, almost half of the sample population was overweight or obese, and 15% of the sample had a high waist circumference. Approximately 51% of the adolescents had at least one component of MetS (low HDL levels, high blood pressure, high fasting glucose, high triglycerides or abdominal obesity), and the prevalence of these components was higher among children with obesity, followed by those with overweight and then those with normal weight. This is a cause of great concern since obesity and metabolic disturbances can remain until adulthood, increasing the risk of diseases later in life [4, 5]. The rate of MetS (as well as all of its biochemical markers) was significantly higher in obese people (16%) than in overweight individuals (2%). This finding is strongly supported in the literature; for instance, among 263 Lebanese adolescents, MetS was identified in approximately 21% of obese, 4% of overweight, and 1% of normal weight subjects [16].

HDL (followed by high blood pressure) was found to be the predominant risk factor among adolescents living in Dubai, as was previously reported in different studies performed in the UAE [31] and other countries in the region [41, 44]. If MetS did not account for HDL levels, our data would have resulted in a prevalence of 0.8% of MetS (only 5 students). This assumption, in addition to the results of the logistic regression, demonstrates the impact of low HDL levels on health.

The presence of genetic determinants for MetS has been suggested, and these could make certain populations more vulnerable to developing chronic abnormalities. Studies that investigate genetic susceptibility to having low HDL have identified several predisposing variants among Arabs [45]. This hypothesis was supported in a recent study that identified many predisposing variants at the genome level that affected the expression of cholesteryl ester transfer protein (CETP) and LIM- and cysteine-rich domain 1 (LMCD1) genes, resulting in low HDL levels in ethnic Arabs [45]. These results were strongly supported by other papers that assessed the lipid profile of all age groups in the region. Bayoumi et al. [44] mentioned that in Omani people, weight and HDL levels were highly determined by genetics, while other risk factors were triggered by environmental influences. Additionally, approximately 87% of Saudi children and adolescents aged between 10 and 18 years old had low HDL levels [41], and this was classified as the most dominant risk factor among the population. Very similar results were also found among Saudi adults [38]. Since low HDL levels are a risk factor of CVDs, this theory could explain why the eastern Mediterranean region [46], which mostly includes Arab countries [7], has the highest prevalence of deaths attributed to CVD.

Despite the fact that there is evidence showing that a genetic component is associated with the low HDL levels observed among Arab populations, it is crucial to look at the picture from different angles. There are several types of HDL cholesterol, and they have different risk levels. For instance, when apolipoprotein A-I Milano (ApoA-1_M_) is the predominant HDL particle, the pro-atherogenic effect of low HDL levels is abated [47]. This was first discovered in Italy, a country that neighbours several Arab countries and contains populations that could share the same genetic inheritance. This hypothesis needs substantial investigation of HDL apolipoproteins among our populations.

Finally, it is crucial to define a gold standard method to determine HDL levels. A study published by Mehairi et al. [31] recently assessed the prevalence of MetS in 1018 adolescents aged 12 to 18 years old living in Al Ain, UAE, and found that the prevalence was 13% when the IDF reference was used.

If we dig deeper into the baseline biochemical characteristics of the sample population, the large variance can be explained. If we exclude blood pressure and waist circumference measurements, which rely on the skills of the data collectors, the differences in biochemical parameters among both populations do not exceed 13% (no differences for LDL or total cholesterol values, while the difference for glucose was approximately 4% and that of triglycerides was 13%) except for HDL values, which varied by 23%. The biochemical blood analyses performed in the two studies took place in different laboratories that used different methods to analyse HDL levels. It is important to note that there is no consensus regarding the analysis of HDL, and differences in the methods used to obtain measurements could result in large variations in results. The higher HDL values reported in the paper by Mehairi et al. [31] could have resulted in a lower prevalence of MetS since low HDL levels were found to be the most predominant risk factor.

### Limitations

Our study has a number of limitations. First, our sample population was drawn from only 1 Emirate: Dubai. The UAE federation consists of 7 Emirates, and although the Emirati populations across the UAE have similar dietary habits, we could not confirm how representative our sample population is of the country as a whole. Second, our sample was drawn only from public/government schools, which mainly consist of Emirati students. However, a small percentage of Emiratis also attend private schools. Hence, the inclusion of private schools may have produced different results. Finally, no waist circumference cut-off values were available for our population; hence, we used international cut-off points.

## Conclusions

In conclusion, the prevalence of MetS among a random representative sample of adolescents between 10 and 15.9 years old who attended public schools in Dubai was 3.7%. Our findings emphasize the importance of early screening interventions for metabolic abnormalities in adolescents, given the risk that children with MetS could develop chronic diseases, such as type 2 diabetes, later in life. This study also showed that low HDL levels were the most prevalent and significant reason for the prevalence of MetS in adolescents, and we propose that there may be genetic susceptibility leading to the low HDL levels among this population. Further investigations are needed to explore this issue, as certain types of apolipoproteins that form HDL particles can protect against the pro-atherogenic effects of low HDL levels. This study also urges for consensus to be reached regarding how serum HDL levels should be measured, since differences in methodologies may lead to substantial variations in results.

## Abbreviations

BMI: Body mass index
CDC: Centers for Disease Control
CETP: Cholesteryl ester transfer protein
CVD: Cardiovascular disease
DBP: Diastolic blood pressure
HDL: High-density lipoprotein
IDF: International Diabetes Federation
LDL: Low-density lipoprotein
LMCD1: LIM- and cysteine-rich domains 1
NCD: Non-communicable disease
NCEP: National Cholesterol Education Program
MetS: Metabolic syndrome
SBP: Systolic blood pressure
SD: Standard deviation
SHBG: Sex hormone-binding globulin
UAE: United Arab Emirates
WHO: World Health Organization

## References

[1] Obeid O (2013). Low phosphorus status might contribute to the onset of obesity. Obes Rev.

[2] Ministry of Health, United Arab Emirates global school-based student health survey 2005. Centers for disease control, World Health Organization; 2005. http://www.who.int/ncds/surveillance/gshs/2005_United_Arab_Emirates_GSHS_Country_Report.pdf

[3] Ministry of Health, United Arab Emirates Global School-based Student Health Survey United Arab Emirates 2010 Fact Sheet. Centers for Disease Control, World Health Organization; 2010. http://www.who.int/ncds/surveillance/gshs/UAE_2010_FS.pdf

[4] Burke V (2005). Predictors of body mass index and associations with cardiovascular risk factors in Australian children: a prospective cohort study. Int J Obes.

[5] Katzmarzyk PT (2001). Stability of indicators of the metabolic syndrome from childhood and adolescence to young adulthood: the Quebec family study. J Clin Epidemiol.

[6] Zimmet P (2007). The metabolic syndrome in children and adolescents–an IDF consensus report. Pediatr Diabetes.

[7] Organization, W.H (2014). Noncommunicable Diseases Country Profiles 2014.

[8] Musaiger AO (2011). Overweight and obesity in eastern mediterranean region: prevalence and possible causes. J Obes.

[9] Lauer R, Clarke W (1989). Childhood risk factors for high adult blood pressure: the Muscatine study. Pediatrics.

[10] He FJ, MacGregor GA (2006). Importance of salt in determining blood pressure in children. Hypertension.

[11] Johnson WD (2009). Prevalence of risk factors for metabolic syndrome in adolescents: National Health and nutrition examination survey (NHANES), 2001-2006. Arch pediatr adolesc med.

[12] Cook S (2003). Prevalence of a metabolic syndrome phenotype in adolescents: findings from the third National Health and nutrition examination survey, 1988-1994. Arch pediatr adolesc med.

[13] Sliem HA (2012). Metabolic syndrome in the Middle East. Indian journal of endocrinology and metabolism.

[14] Al-Daghri NM (2014). Gender-dependent associations between socioeconomic status and metabolic syndrome: a cross-sectional study in the adult Saudi population. BMC Cardiovasc Disord.

[15] Khader Y (2007). High prevalence of the metabolic syndrome among northern Jordanians. J Diabetes Complicat.

[16] Nasreddine L (2012). Obesity is associated with insulin resistance and components of the metabolic syndrome in Lebanese adolescents. Ann Hum Biol.

[17] Ella NAA (2010). Prevalence of metabolic syndrome and insulin resistance among Egyptian adolescents 10 to 18 years of age. J Clin Lipidol.

[18] Malik M, Razig SA (2008). The prevalence of the metabolic syndrome among the multiethnic population of the United Arab Emirates: a report of a national survey. Metab Syndr Relat Disord.

[19] Al-Sarraj T (2010). Metabolic syndrome prevalence, dietary intake, and cardiovascular risk profile among overweight and obese adults 18–50 years old from the United Arab Emirates. Metab Syndr Relat Disord.

[20] Malik M, Bakir A (2007). Prevalence of overweight and obesity among children in the United Arab Emirates. Obes Rev.

[21] Alsafar H (2012). The prevalence of type 2 diabetes mellitus in the United Arab Emirates: justification for the establishment of the emirates family registry. International Journal of Diabetes in Developing Countries.

[22] Al Amiri E (2015). The prevalence, risk factors, and screening measure for prediabetes and diabetes among Emirati overweight/obese children and adolescents. BMC Public Health.

[23] Kuczmarski, R.J., et al., 2000 CDC Growth Charts for the United States: methods and development*.* Vital and health statistics. Series 11, Data from the national health survey, 2002(246): p. 1–190. 12043359

[24] Organization, W.H (1999). Diagnosis and classification of diabetes mellitus and its complications: report of a WHO consultation.

[25] Balkau B, Charles M-A (1999). Comment on the provisional report from the WHO consultation. Diabet Med.

[26] Expert Panel on Detection, E (2001). Executive summary of the third report of the National Cholesterol Education Program (NCEP) expert panel on detection, evaluation, and treatment of high blood cholesterol in adults (adult treatment panel III). Jama.

[27] Goodman N (2001). American Association of Clinical Endocrinologists medical guidelines for the clinical practice for the diagnosis and treatment of hyperandrogenic disorders. Endocr Pract.

[28] Alberti KGMM, Zimmet P, Shaw J (2006). Metabolic syndrome—a new world-wide definition. A consensus statement from the international diabetes federation. Diabet Med.

[29] Mancini MC (2009). Metabolic syndrome in children and adolescents-criteria for diagnosis. Diabetology & metabolic syndrome.

[30] Hajat C, Shather Z (2012). Prevalence of metabolic syndrome and prediction of diabetes using IDF versus ATPIII criteria in a Middle East population. Diabetes Res Clin Pract.

[31] Mehairi AE (2013). Metabolic syndrome among Emirati adolescents: a school-based study. PLoS One.

[32] Aziz F., Al Maskari F., Shah S. M. (2015). Metabolic Syndrome Among Healthy Children Aged 6 to 12 Years in Al Ain, United Arab Emirates. PEDIATRICS.

[33] Al Dhaheri AS (2016). A cross-sectional study of the prevalence of metabolic syndrome among young female Emirati adults. PLoS One.

[34] Al-Isa A, Akanji AO, Thalib L (2010). Prevalence of the metabolic syndrome among female Kuwaiti adolescents using two different criteria. Br J Nutr.

[35] Sangun Ö (2011). Prevalence of metabolic syndrome in obese children and adolescents using three different criteria and evaluation of risk factors. J Clin Res Pediatr Endocrinol.

[36] Ahmadi A (2013). Metabolic syndrome in Iranian youths: a population-based study on junior and high schools students in rural and urban areas. J diabetes res.

[37] Khashayar P (2013). Metabolic syndrome and cardiovascular risk factors in a national sample of adolescent population in the middle east and North Africa: the CASPIAN III study. Int J Endocrinol.

[38] Al-Daghri NM (2010). Decreasing prevalence of the full metabolic syndrome but a persistently high prevalence of dyslipidemia among adult Arabs. PLoS One.

[39] Agirbasli M (2006). Metabolic syndrome in Turkish children and adolescents. Metabolism.

[40] Mirhosseini N-Z (2009). Prevalence of the metabolic syndrome and its influencing factors among adolescent girls in Mashhad, Iran. Asia Pac J Clin Nutr.

[41] Al-Daghri NM (2010). Extremely high prevalence of metabolic syndrome manifestations among Arab youth: a call for early intervention. Eur J Clin Investig.

[42] Mehrkash M (2012). Obesity and metabolic syndrome among a representative sample of Iranian adolescents. Southeast Asian J Trop Med Public Health.

[43] Agirbasli M (2009). Sex hormones and metabolic syndrome in children and adolescents. Metabolism.

[44] Bayoumi RA (2007). Heritability of determinants of the metabolic syndrome among healthy Arabs of the Oman family study. Obesity.

[45] Wakil SM (2016). A common variant association study reveals novel susceptibility loci for low HDL-cholesterol levels in ethnic Arabs. Clin Genet.

[46] Ng SW (2011). The prevalence and trends of overweight, obesity and nutrition-related non-communicable diseases in the Arabian Gulf States. Obes Rev.

[47] Franceschini G (1980). A-IMilano apoprotein. Decreased high density lipoprotein cholesterol levels with significant lipoprotein modifications and without clinical atherosclerosis in an Italian family. J Clin Investig.

